# Potential technique for improving the survival of victims of tsunamis

**DOI:** 10.1371/journal.pone.0197498

**Published:** 2018-05-23

**Authors:** Akane Kurisu, Hisami Suga, Zdenek Prochazka, Kojiro Suzuki, Kazumasa Oguri, Tetsunori Inoue

**Affiliations:** 1 Akatutumi, Setagayaku, Tokyo, Japan; 2 Department of Biogeochemistry, Japan Agency for Marine-Earth Science and Technology, Yokosuka, Kanagawa, Japan; 3 Department of Information Engineering, National Institute of Technology, Oita College, Oita, Oita, Japan; 4 Coastal and Ocean Engineering Department, Port and Airport Research Institute, Yokosuka, Kanagawa, Japan; 5 Department of Marine Biodiversity, Japan Agency for Marine-Earth Science and Technology, Yokosuka, Kanagawa, Japan; 6 Marine Information and Tsunami Department, Port and Airport Research Institute, Yokosuka, Kanagawa, Japan; Tianjin University, CHINA

## Abstract

We investigated a method for surviving tsunamis that involved the use of personal flotation devices (PFDs). In our work, we succeeded in numerically demonstrating that the heads of all the dummies wearing PFDs remained on the surface and were not dragged underwater after the artificial tsunami wave hit them. In contrast, the heads of all the dummies not wearing PFDs were drawn underwater immediately; these dummies were subsequently entrapped in a vortex. The results of our series of experiments are important as a first step to preventing the tragedies caused by tsunamis.

## Introduction

According to the definition given by the World Health Organization (WHO), “tsunamis are giant sea waves that are produced by submarine earthquake or slope collapse into the seabed” [1]. During the 21st century, the world has already experienced at least two tremendous earthquakes, namely, the Sumatra-Andaman Earthquake (M_W_ 9.1) on December 26, 2004, and the Tohoku-Pacific Earthquake (M_W_ 9.0) on March 11, 2011 [2,3]. Both of these earthquakes caused devastating tsunamis that claimed the lives of approximately 230,000 and 18,000 people, respectively. It is very important to study the mechanisms of tsunamis and how they impact people in order to prevent heavy casualties during the next huge tsunami, which is expected to occur in the near future [4–9]. While there are papers and websites that discuss how to evacuate safely during a tsunami [10,11], to the best of our knowledge, there are no papers describing how people move in the water when engulfed in a tsunami. Some websites say that personal floatation devices (PFDs) are effective when dealing with a tsunami, but the grounds for this statement are not clear [12].

Past tsunami disasters have shown us that it is extremely difficult even for good swimmers to escape from drowning. According to the new definition adopted by the WHO in 2002, “drowning is the process of experiencing respiratory impairment from submersion/immersion in liquid” [13]. If a victim cannot rise to the surface of the water, the victim will lose consciousness within a short period of time, and then, breathing will stop and cardiac arrest will follow within 4–5 min [14].

To study why this is so, it is necessary to survey the movements of people after they are swallowed into a tsunami. Furthermore, it would be of great interest to investigate whether a person who wears a PFD would have a greater chance of survival. When people without PFDs are dragged under the water and cannot resurface, they will not be able to hold their breath and will immediately start gasping for air, which will be followed by rapid, deep breaths and an increase in breathing volume to about five times that of the normal resting level [15,16]. In our work, we have conducted a series of experiments at a large flume and observed the movements of dummies with and without PFDs attached to them during a simulated tsunami.

Here, we demonstrate numerically that the heads of all the dummies wearing PFDs remained on the surface and were never dragged down into the water after being struck by artificial tsunami waves. In contrast, the heads of all the dummies not wearing PFDs were drawn underwater and entrapped in a vortex immediately. The results of this series of experiments are an important first step for improving tsunami survivorship during future disasters.

## Methods

### Large Hydro Geo Flume

**Fig 1:**
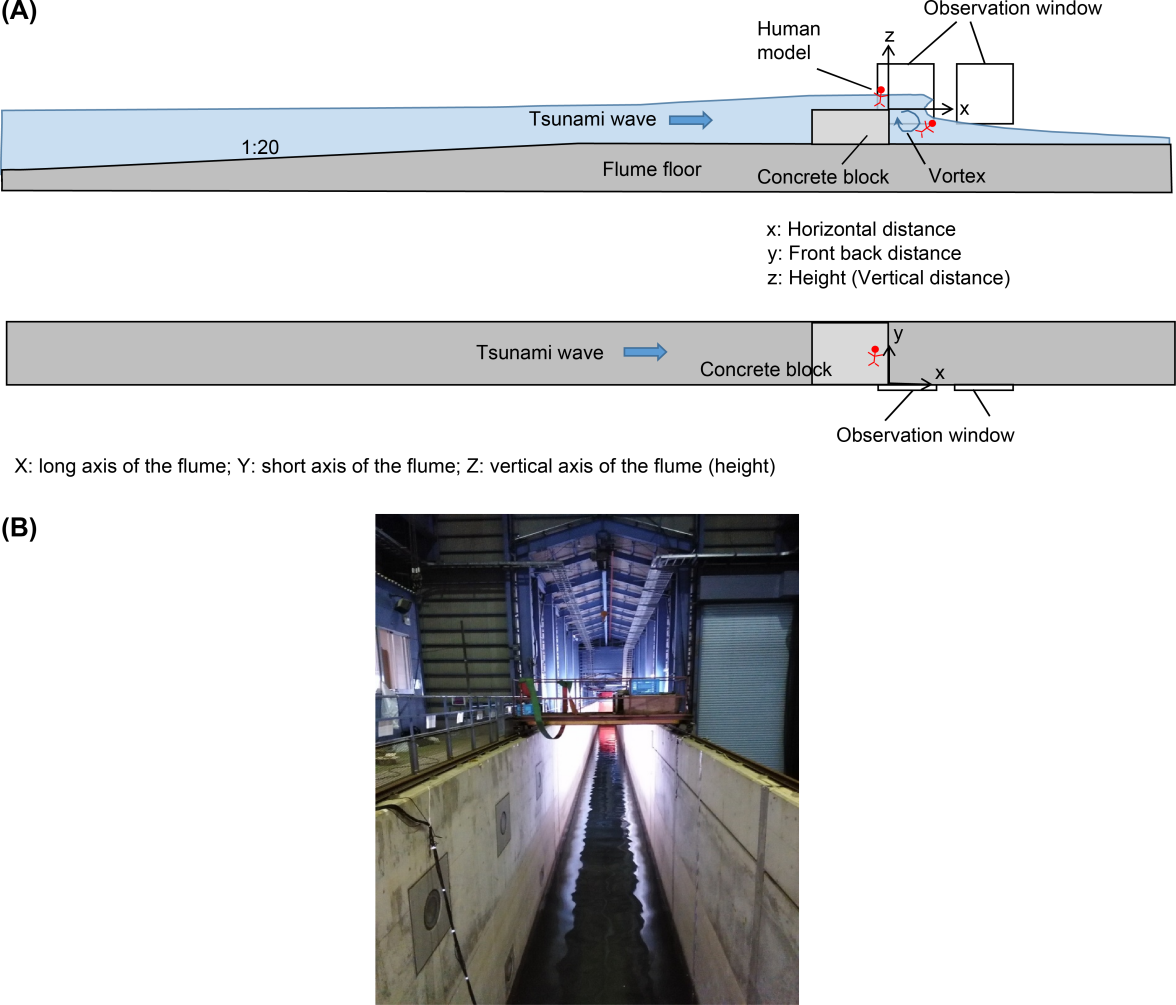
(A) Side-view and top-view schema and (B) Overhead view of the Large Hydro Geo Flume.

Experiments were conducted at the Large Hydro Geo Flume in the Port and Airport Research Institute, Nagase, Yokosuka, Kanagawa Prefecture, Japan. This flume is 184 m long, 3.5 m wide, and 12 m deep, and it has two side windows (every window is 2.7 m in length and 2.5 m in height) that enable observers to view the sides of the pool water (Fig 1A and 1B) [17].

Simulated tsunami waves (0.59 ± 0.13 m high) hit the dummies within the flume.

### Dummies

**Fig 2:**
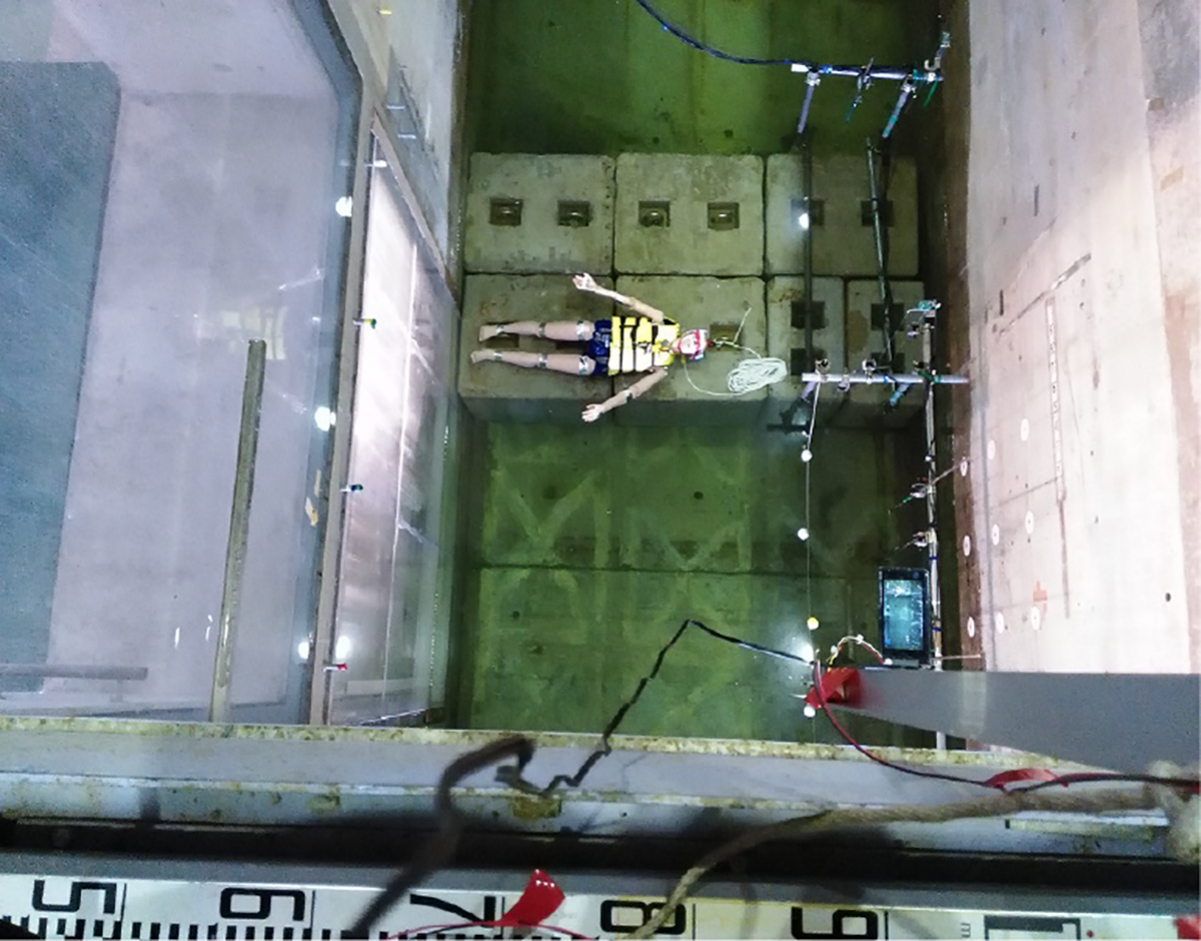
Dummy placed above the concrete block at the bottom of the flume.

The dummies employed in this study were Simulaids’s® Water Rescue Manikin (Item number 1328). In every experiment, the internal cavity of the dummy was filled with water to maintain its weight in air at about 48 kg and its specific gravity at 1.05, which is almost equal to a human’s specific gravity. A light-emitting diode was installed on the head of every dummy to facilitate tracking of the positions of the dummy. Every dummy was lowered down to the concrete block on the floor of the flume. Then, the dummy was positioned on the concrete block at right angles to the long axis of the flume in a supine position. The top of the block was 20 cm below the water surface (Fig 2).

### Personal flotation devices (PFDs)

The PFDs employed in this study were Marlin Australia PTY LTD’s Model No. 2220, General Purpose Vest, ADULT Universal, Type III, U.S. Coast Guard Approval No: 160.064/4507/0. The PFDs employed had a buoyancy of 7.031 kg (15.5 lbs).

### Calculation of the positions of the heads of dummies

Video sequences of the dummies carried by tsunami waves were recorded by using two synchronized cameras. The first camera was used to capture a side view, and the other one was used to capture a top view. Recorded video sequences were split into separate image frames, where the frame rate was reduced to 1/10 of each original sequence. Next, the position of the head of the dummy was manually marked in each image frame, and the pixel coordinates of the marks were extracted by using an image processing method. Extracted pixel coordinates were then used to estimate the position of the dummy’s head in the real coordinate system. The horizontal position of the dummy’s head was calculated from the pixel position in each top-view image, where the perspective distortion was not taken into consideration. On the other hand, the water depth above the head, i.e., vertical distance between water level and head position, was calculated from the pixel position in each side-view image. These calculations were performed with respect to the perspective distortion, i.e., the distance from the side-view camera was used to correct the perspective distortion of the water depth above the dummy’s head. The distance from the side-view camera was acquired from the top-view images [18].

## Results

A series of experiments were conducted at the Large Hydro Geo Flume in the Port and Airport Research Institute from June 27, 2016, to June 30, 2016. Experiments were planned to evaluate the movements of dummies just after a tsunami strike.

The experiments were divided into the following two groups: (1) group with PFDs (Experiment Nos. 1–4) and (2) group without PFDs (Experiment Nos. 5–10).

**Fig 3:**
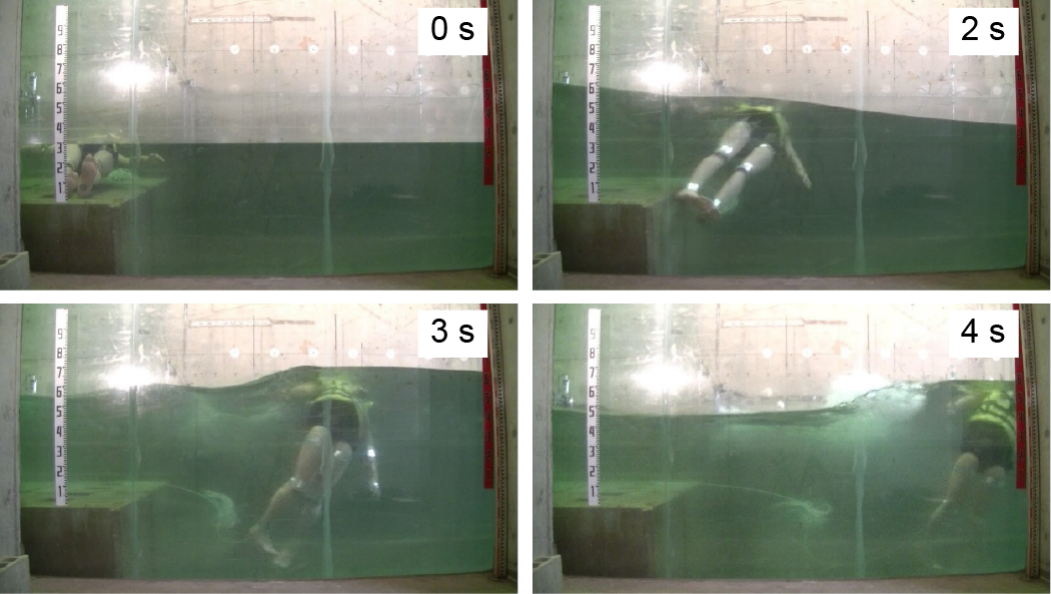
Frames from S1 Video, Experiment No. 2 (side view, dummy with a PFD).

**Fig 4:**
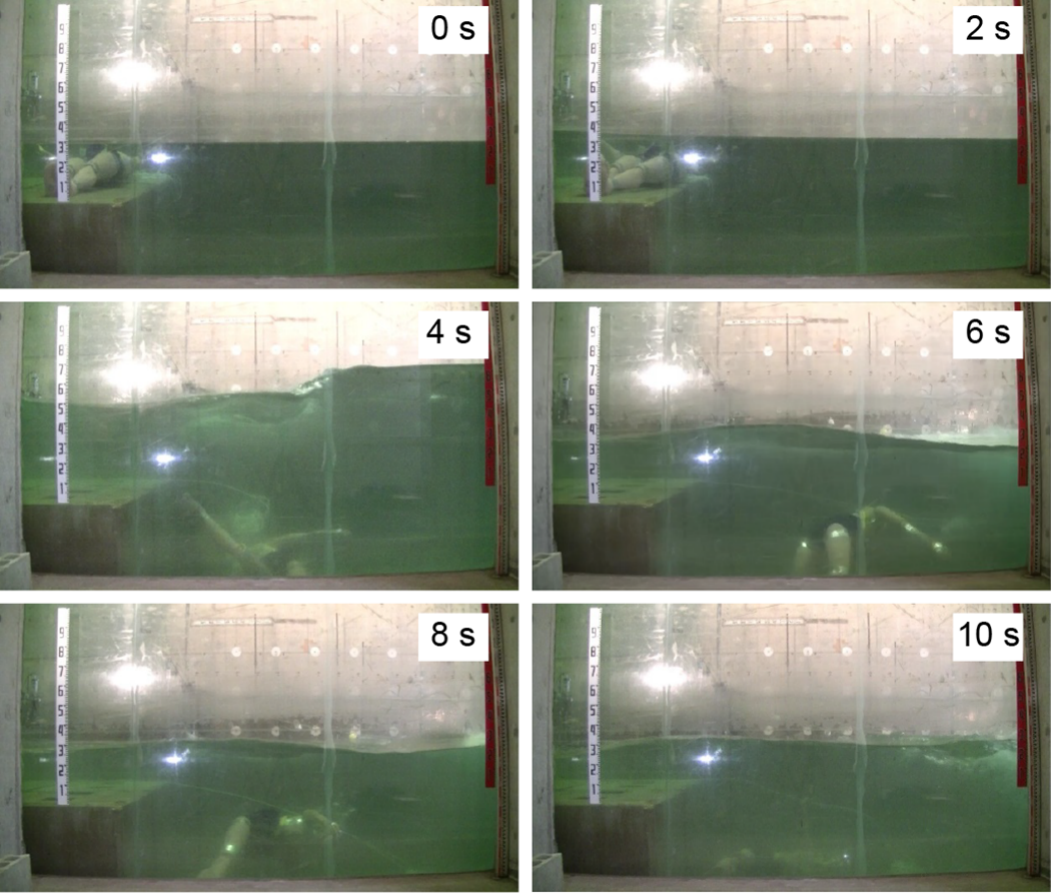
Frames from S2 Video, Experiment No. 6 (side view, dummy without a PFD).

**Fig 5:**
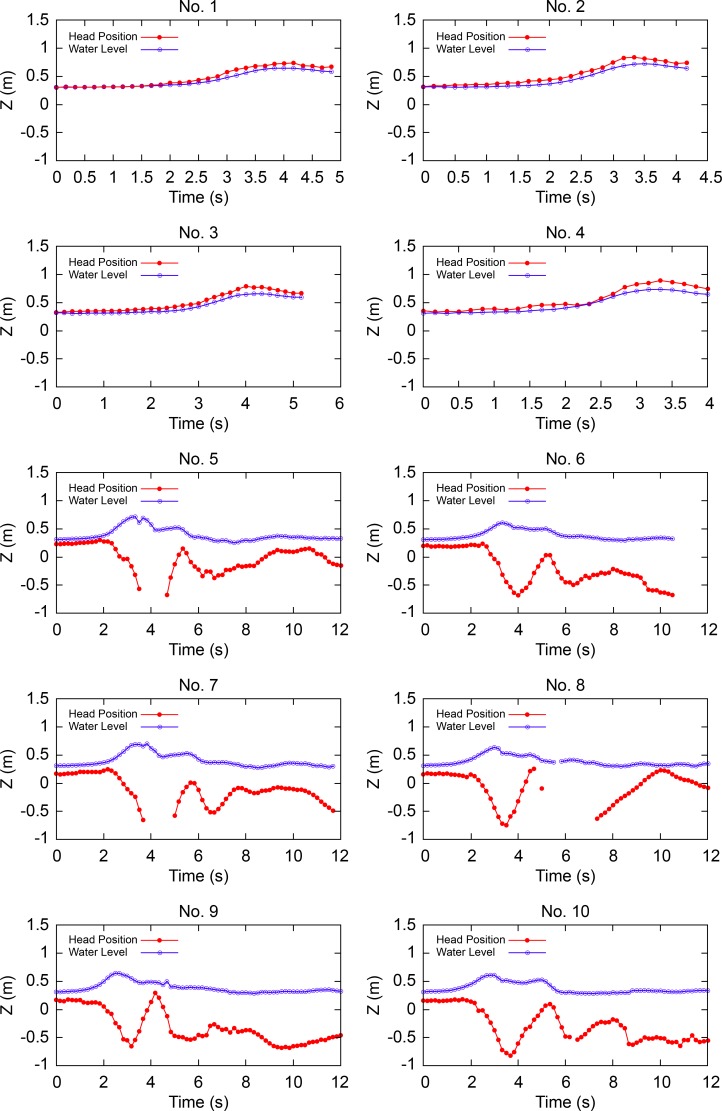
Side view of the movement of dummies with and without PFDs.

This series of experiments revealed several key findings. Example side-view images of the movement of dummies with and without PFDs are shown in Figs 3 and 4, respectively. First, after the tsunami struck, the heads of all the dummies wearing PFDs remained afloat; the heads were higher than the water level and were never dragged underwater. The dummies with PFDs were carried in motions similar to that of surfers during wave surfing (Figs 3 and 5, No. 1, 2, 3, 4).

Conversely, the heads of all the dummies not wearing PFDs were entrapped in vortices about 2 s after the tsunami wave hit them. Then, they sank to the deepest level and rose again to -0.3 ± 0.1 m. Afterward, they continued whirling intensively up and down in the water but never came up to the water surface (Figs 4 and 5, No. 5, 6, 7, 8, 9, 10). The No. 5, 7, and 8 dummies sank too deep to estimate the positions of their heads at certain time points.

**Fig 6:**
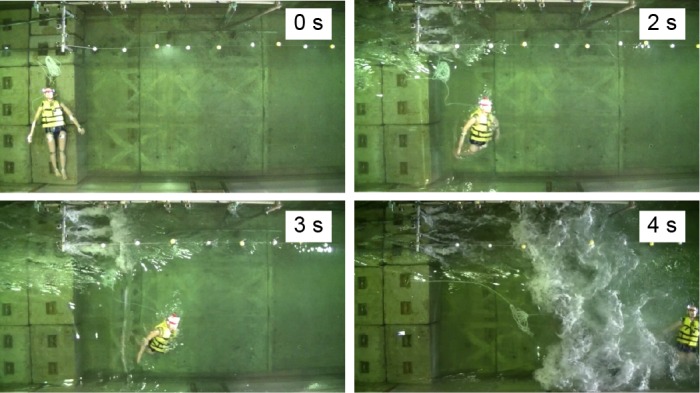
Frames from S3 Video, Experiment No. 2 (top view, dummy with a PFD).

**Fig 7:**
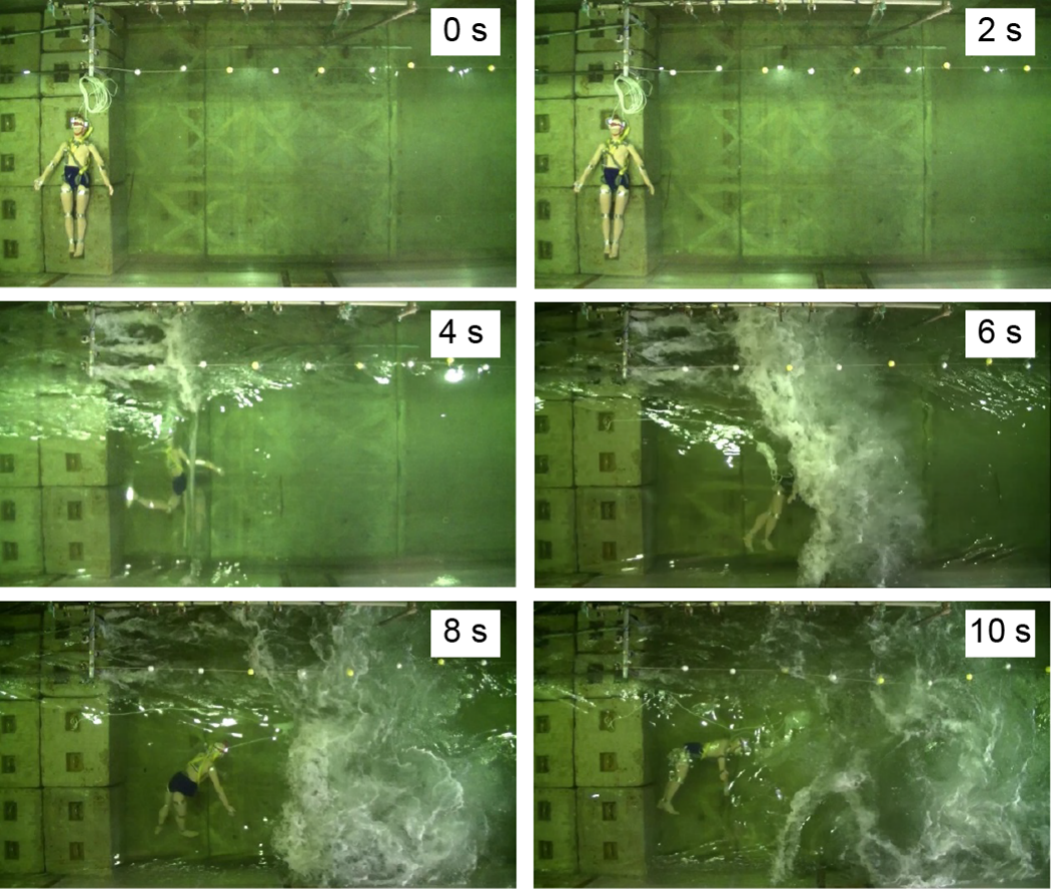
Frames from S4 Video, Experiment No. 6 (top view, dummy without a PFD).

**Fig 8:**
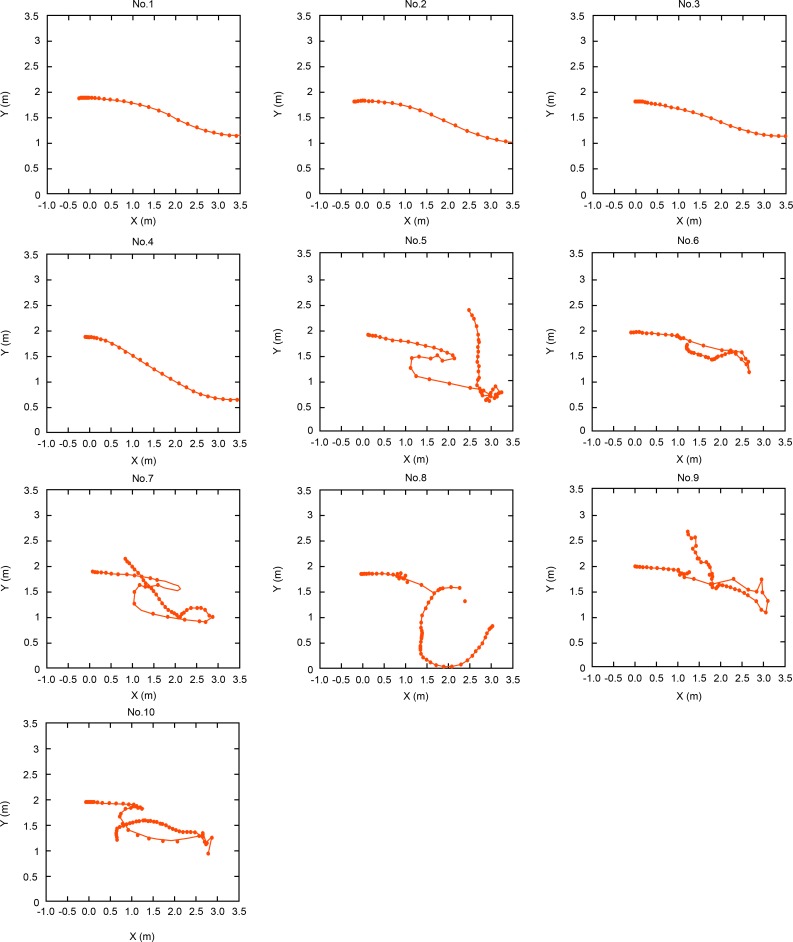
Top view of the movement of dummies with and without PFDs.

Example top-view images of the movement of dummies with and without PFDs are shown in Figs 6 and 7, respectively. The top-view data show that the heads of all the dummies wearing PFDs moved away in a softly curving path (Figs 6 and 8, No. 1, 2, 3, 4). In contrast, the heads of all the dummies not wearing PFDs remained within 3.5 m from the starting point and displayed irregular movements (Figs 7 and 8, No. 5, 6, 7, 8, 9, 10).

**Fig 9:**
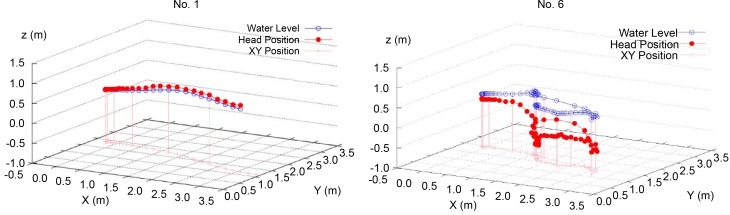
Three-dimensional movements of the dummies’ heads in Experiment No. 1 and 6.

The movements of the dummies’ heads in Experiment No. 1 and 6 are shown three-dimensionally in Fig 9. In Experiment No. 1 (with a PFD), the dummy moved on the surface of the water. Conversely, in Experiment No. 6 (without a PFD), the dummy was whirling in the water.

## Discussion

A typical tsunami has a very high speed of roughly 700 km/h as it emerges from the deep sea, after which it suddenly slows down when it reaches shallow coastal regions where it may retain a relatively high speed of about 40 km/h. At these moments, tsunami waves rear up precipitously and one can realize the Japanese meaning of the word “tsunami” (wave “nami” in a harbor “tsu”). This is when people witness giant surges; tsunami waves will often overcome a majority of witnesses, even if they run away to save their lives. In addition, when a tsunami recedes, the water will sweep away victims and debris from the land to the sea [19]. As recommended by the Japanese Sanriku coast’s old adage “Tsunami tendenko” (“Run uphill on your own will when a tsunami comes”), the first defensive action against any tsunami is to act quickly and seek higher ground immediately. However, since a highly reliable tsunami detection and warning system is still in the developmental stages, people are apt to delay or even ignore evacuations [20–22]. Some of them even deliberately take the opposite direction and head towards the beach to watch the incoming tsunami [23].

Findings from the systematic literature review indicate that the primary cause of tsunami-related mortality is drowning [24]. During the 2004 Sumatra-Andaman Earthquake, the main cause of death was drowning due to the tsunami [25–27]. According to the Japanese National Police Agency’s report based on data obtained from post-mortem certificates after the Tohoku-Pacific Earthquake, the cause of death for 14,308 of the 15,786 fatalities (90.64%) was drowning, while 667 (4.23%) died from severe impact injuries [28].

People might be crushed to death by various debris such as that from destroyed houses and buildings, wrecked boats, and cars, which would be whirling in the water, or they might be crushed against a wharf or breakwater and suffer fatal injuries. Even so, we have to admit that large numbers of people were engulfed in the tsunami waves and drowned in the Tohoku-Pacific Earthquake on March 11, 2011. Therefore, there is an urgent need to find a technique that can prevent drowning. With such a technique, it would be possible to reduce the number of victims who drown and die in tsunamis. Unfortunately, however, there is a lack of information on the cause of drowning during tsunami disasters. During the Tohoku-Pacific Earthquake, the body of every victim of the tsunami was examined by a forensic doctor or a medical coroner, and through this examination, it was confirmed that the cause of death of almost all the victims was drowning. However, there were no detailed descriptions beyond drowning in the post-mortem certificates. Why could the tsunami victims not swim? Why were they not able to cling firmly to floating objects on the surface of water? We searched the literature and could not find answers to these important questions. Hence, we conducted a series of experiments to analyze the cause of drowning during a tsunami.

In our experiments, all the heads of dummies not wearing PFDs were entrapped in vortices after the tsunami wave hit them. They continued whirling intensively up and down in the water but never came up to the water surface.

When tsunamis engulf people below the water surface, and the rate as well as the depth of their breathing increase dramatically, people have no other choice but to inhale water since it would be nearly impossible to swim up to the water surface. This will greatly increase the risk of drowning. Since tsunamis, whose wavelengths are very long, are generated by the displacement of huge volumes of water, the whirlpools created by them are extremely powerful and continue for a long time. Therefore, once people without PFDs are caught up in a tsunami, it is very difficult for them to escape from such whirlpools. Even skilled swimmers without PFDs would not be able to resurface quickly and remain afloat. This severe whirling of tsunami waves is likely one of the main factors that cause the overwhelming majority of tsunami victims to drown.

The buoyancy of widely popular PFDs is 7.031 kg (15.5 lbs) [29], and the effectiveness of PFDs has been studied for recreational swimming and fishing [30–32]. However, to the best of the authors’ knowledge, there is a lack of information on the effectiveness of PFDs during catastrophic tsunami disasters. In our experiments that employed widely popular PFDs, the dummies wearing the PFDs were not dragged underwater. They remained afloat and the heads were higher than the water level.

As our experiments demonstrated, it can be concluded that when people are engulfed within tsunami waves, PFDs will provide them with a higher chance of survival because they will remain on the surface of tsunami waves and are still able to breathe. In other words, a PFD is a critical piece of equipment for surviving tsunamis. In critical situations, when a tsunami wave is already visible to people and there are no PFDs around, they might be able to put empty plastic bottles between their skin and clothing, hold on to garbage cans, or wear helmets as substitutions for PFDs and other protective gear. These actions represent the second-best tsunami survival technique.

It is reasonable to assume that hypothermia victims were included in the 15,786 fatalities (90.64%) of the Tohoku-Pacific Earthquake because the seawater temperature was very low (5 to 7°C) [33]. If the entire human body is immersed in water at such low temperatures, its core temperature will decrease to a critical level within 2 h [34]. People with PFDs might be transported extensively far from the coast; therefore, it will be necessary to establish a reliable rescue system to save them before their core temperature drops to a severe level where hypothermia can set in. People lose consciousness when their core temperature drops to 30°C. However, if people wear PFDs, they would be able to avoid drowning even if they lose consciousness as they would merely float on the water surface while still retaining their ability to breathe [34–36].

Tsunamis might kill people in multiple ways as mentioned above. In the next series of experiments, we plan on evaluating whether a dummy wearing a PFD would be able to overcome a crash with debris swept by the water or a crash against a concrete block.

Notably, the wave heights of our artificial tsunamis were much lower than those of natural tsunamis, which often exceed 10 m. Regardless, our experiments demonstrated that dummies without PFDs were caught up in the vortex and could not resurface after they were hit by the artificial tsunami. On the other hand, dummies wearing PFDs were not drawn under and were able to continue to float on the water surface. Based on these results, we are planning to carry out further experiments with a 1.5 m high artificial tsunami and simulations of 10 m high tsunami waves using computer software [37,38].

The results of our series of experiments are important as a first step to improve survivorship during tsunami disasters, and application of the results could likely save numerous lives.

## Conclusion

Our experiments with approximately 50 cm high artificial tsunami waves demonstrated that PFD use is an effective technique to prevent drowning during a tsunami. Specifically, the heads of all the dummies not wearing PFDs were entrapped in a vortex and drawn underwater immediately. In contrast, the heads of all the dummies wearing standard PFDs remained on the surface and were not dragged underwater. These findings were obtained through video images, which were taken from the side window of a large flume.

Drowning is the main cause of death during a tsunami. Thus, use of PFDs during a tsunami could potentially save numerous lives.

## Supporting information

S1 Video Experiment No. 2. Dummy with a PFD, side view.
(MP4)

S2 Video Experiment No. 6. Dummy without a PFD, side view.
(MP4)

S3 Video Experiment No. 2. Dummy with a PFD, top view.
(MP4)

S4 Video Experiment No. 6. Dummy without a PFD, top view.
(MP4)
